# Biometrics Assessment of Cluster- and Berry-Related Traits of Muscadine Grape Population

**DOI:** 10.3390/plants10061067

**Published:** 2021-05-26

**Authors:** Jiovan Campbell, Ali Sarkhosh, Fariborz Habibi, Ahmed Ismail, Pranavkumar Gajjar, Ren Zhongbo, Violeta Tsolova, Islam El-Sharkawy

**Affiliations:** 1 Center for Viticulture and Small Fruit Research, College of Agriculture and Food Sciences, Florida A&M University, Tallahassee, FL 32308, USA; jiovan1.campbell@famu.edu (J.C.); ahmed.ismail@agr.dmu.edu.eg (A.I.); pranavkumar1.gajjar@famu.edu (P.G.); zhongbo.ren@famu.edu (R.Z.); violeta.tsolova@famu.edu (V.T.); 2 Horticultural Sciences Department, University of Florida, Gainesville, FL 32611, USA; sarkhosha@ufl.edu; 3 Department of Horticultural Science, School of Agriculture, Shiraz University, Shiraz 71441-65186, Iran; fariborz_h659@yahoo.com; 4 Department of Horticulture, Faculty of Agriculture, Damanhour University, Damanhour P.O. Box 22516, Egypt

**Keywords:** biometrics, berry-related traits, cluster-related traits, flower structure, muscadine grape

## Abstract

In this study, biometrics assessment of flower structure, cluster-, and berry-related traits were evaluated in a population of 90 muscadine grape genotypes for three consecutive years. This population consisted of 21 standard cultivars, 60 breeding lines, and 9 *Vitis x Muscadinia* hybrids (VM hybrids). Cluster length (CL) and width (CWI) characteristics exhibited slight differences among the population, with a range estimated at 7.1 and 4.6 cm, respectively. However, cluster weight (CWE), number of berries/cluster (N.B/C), and cluster compactness (CC) traits showed more diversity between individuals with a calculated range of 205.6 g, 32.6 B/C, and 24.1, respectively. Interestingly, all berry-related traits greatly varied between individuals, excluding the number of seeds/berry (N.S/B) character. The N.S/B trait displayed a narrow range of 5.6 seeds within the population. However, characters of berry length (BL), width (BWI), weight (BWE), the weight of seeds/berry (W.S/B), firmness (FF), and dry scar pattern (SP) demonstrated a wide estimated range of 21.2 mm, 21.7 mm, 25.4 g, 0.71 g, 0.21 N, and 82%, respectively. Normal distribution analysis for each trait suggested different distribution patterns extended between unimodal to multimodal behavior. Hierarchical mapping analysis was able to classify the population into several clades based on physical cluster- and berry-related attributes. The PCA suggested that hermaphroditic (perfect) flower structure is associated with compact clusters exhibiting small berries in size and weight (i.e., muscadine genotypes suitable for wine production). However, female flower structure is associated with clusters displaying large berries in size and weight (i.e., muscadine genotypes appropriate for fresh consumption). These patterns occurred independently of cluster size and weight characters. This research is the first study evaluating muscadine biometrics characters at a population level, providing valuable information for market demand and muscadine breeding programs.

## 1. Introduction

The grapevine, exploited for the production of wine, table grapes, and raisins, is one of the most widely grown fruit trees. The world production of grapes has registered a notable upward trend during the last decade [1]. Furthermore, the world human consumption of fresh grapes has increased significantly year-on-year, which attracts market interest. The production of table grape cultivars with sensory characteristics highly appreciated by consumers constitutes a major concern for breeding, and it is essential for the highly competitive market. In fresh fruits, visual attributes of table grapes, their chemical constituents, and mechanical properties are involved in consumer acceptability because they are correlated to sensory perception [2,3,4,5,6].

Muscadines and bunch grapes are classified under *Euvitis* genera. However, the pronounced differences in their morphological, phenomic, and genomic characteristics represented by the dissimilarities in stress responses, horticultural and reproductive growth characteristics, and genome structure enabled us to classify them into two different genera, *Muscadinia* and *Vitis* [7]. The most pronounced genetic difference between these two taxa is the number of chromosomes. *Muscadinia* species have 40 chromosomes (*n* = 20); however, bunch grapes (*Vitis* sp.) have 38 chromosomes (*n* = 19). *Muscadinia* also morphologically differs from *Vitis* in their seed, bark, tendril, and cluster structure. Finally, *Muscadinia* displays more tolerance to grape biotic and abiotic stresses [7,8], which prompted efforts to exchange traits via crossing *M. rotundifolia* with *V. vinifera*. Although *Vitis* species hybridize freely, *Vitis x Muscadinia* crosses are complicated, and hybrids are rare and generally sterile, with 39 chromosomes. The morphological differences and similarities are congruous with the biometric results [9,10]. Accordingly, muscadine has challenges that are obviously different from those of traditional bunch grapes. The previous analysis justifies the importance of the current study and highlights muscadine as valuable germplasm that can cause a revolution in grape biology.

Progressively, quantitative approaches to plant morphology are changing the landscape of research for plant ecology, physiology, and evolution. Advances in microscopy, imaging, and computational analyses potentially allow more detailed investigations than have previously been possible. The technology has increased the variety and quantity of data available for phenotypic analysis. It has inspired new directions and applications in the study of plant phenomics. The enriched speed and details that these new technologies can capture suggested that they could potentially be as rich as a bioinformatic data source [11]. As increasingly morphological and image-based data are gathered, the ability to analyze and interpret that data is of the utmost importance. In an age when it is possible to generate the whole genomes sequence, objective, reproducible, and accurate assessments of morphology are a critical missing link to support phenomics and provide a broader context of how the plant morphology relates to ecology, physiology, genotype, and evolutionary and phylogenetic history [11].

It is crucial to understand the physiology of plant development and the impacts of biotic/abiotic stresses on their performance. Therefore, the management practices can be adopted to improve plant/tree productivity and sustainability [12]. Plant biometrics and anatomical measurements help to understand the differences in the growth of plants cultivated in diverse environments. Biometric identification is a pattern of recognition-based classification system that distinguishes an individual by defining its authenticity using a specific physiological or behavioral characteristic.

The biometric system that has been developed during this study is well adapted to facilitate the identification of future muscadine cultivars. Whatever the biometric is defined, it should be universal (i.e., each individual should have that biometric), distinctive (i.e., biometric should be unique to each individual), permanent (i.e., constant over time), and collectible (i.e., can be measured quantitatively) [13]. Living plant recognition is a promising but challenging task in the field of pattern recognition. Therefore, to overcome such a deficit in the representation of muscadine grapes prior research, this study proposes a biometric analysis that composes morphological information, including cluster- and berry-related traits, with multivariate statistical analysis.

Comparative study on biometrics traits of muscadine grapes can provide valuable information for market demand and muscadine breeding program. Therefore, the objective of this study was to determine and compare cluster- and berry-related traits of 90 muscadine genotypes grown in Florida. This research will provide substantial fundamental knowledge to express muscadine grape characteristics and create a classification about biometrics quality attribute for the viticulture industry.

## 2. Results

### 2.1. Determination of Flower Structure (FLS) in Muscadine Population

**Figure 1:**
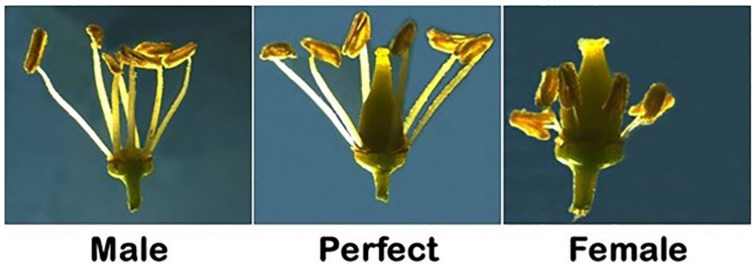
Close up view of staminate (male; **left**), hermaphroditic (perfect; **center**), and imperfect (female, **right**) muscadine flowers collected from O24-10-2, Noble, and Onyx genotypes, respectively.

Native muscadines are typically dioecious [14]. Accordingly, muscadine vines may be male (M) or female (F), although a few naturally occurring hermaphrodite (H) vines have been described [15]. The staminate male flowers have only filaments and anthers, and the flower has merely a whorl of erect stamens containing pollen (Figure 1). The perfect hermaphroditic muscadine flower consists of a typical pistil surrounded by five or more tall, erect stamens producing functional pollen. The filaments are equal or exceed the combined length of the ovary, style, and stigma (Figure 1). The female flowers are imperfect hermaphrodites. The ovary, style, and stigma are thickened and enlarged; however, stamens are both recurved and shorter, and the pollens are sterile (Figure 1). Evaluating the muscadine population for the type of flower indicated that 68.9% (62 genotypes) have hermaphroditic flowers, and 31.1% (28 genotypes) produce female flowers. However, no male muscadine vines were included in this study.

### 2.2. Cluster-Related Traits

#### 2.2.1. Cluster Length

**Figure 2:**
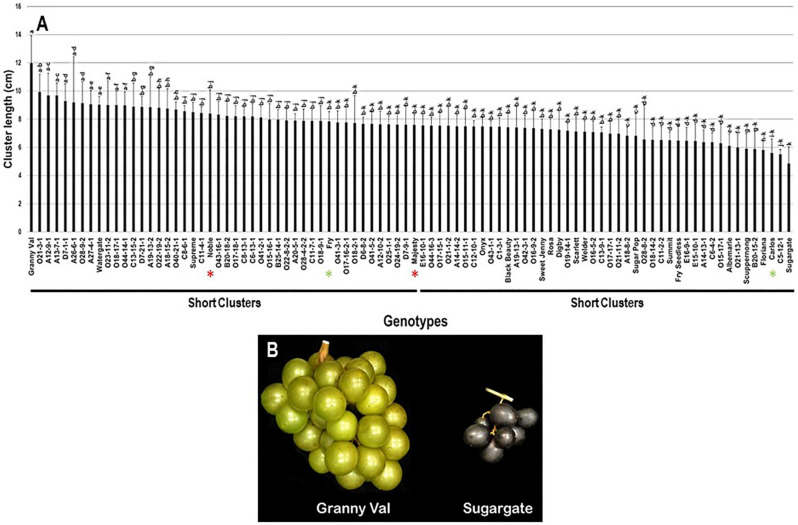
(**A**) Characterization of cluster length (CL) trait among muscadine population (*n* = 90). The bars represent the mean cluster length (±SD) results from five biological replicates among three years (*n* = 15). The y-axis refers to the cluster length (cm), and the x-axis represents the muscadine genotypes. Means within columns for the same letter followed by different letters differ significantly by Tukey’s test (*p* < 0.05). Based on median cluster length (~7.6 cm), the population was divided into two equal groups of long and short clusters. The asterisk refers to the standard commercial colored (red) and bronze (green) cultivars selected as controls in the current study, including Noble, Carlos, Majesty, and Fry. (**B**) A representative image of muscadine genotypes exhibiting the longest (Granny Val) and shortest (Sugargate) clusters.

The cluster length (CL) trait showed a slight diversity among the population with a range of 7.1 cm, by which Granny Val cultivar exhibited the most extended clusters (12.0 cm ± 2.0). In contrast, the Sugargate cultivar displayed the shortest cluster (4.9 cm ± 1.2). The estimated CL average among the population was 7.7 cm ± 1.1. Based on the median CL (~7.6 cm), the population was split into two equal groups of long and short clusters. Accordingly, the standard commercial cultivars Noble (8.4 cm ± 1.7), Fry (7.8 cm ± 0.7), and Majesty (7.6 cm ± 0.7) were classified among the muscadine genotypes exhibiting long clusters. However, Carlos (5.6 cm ± 1.0) belonged to the group displaying short clusters (Figure 2).

#### 2.2.2. Cluster Width

**Figure 3:**
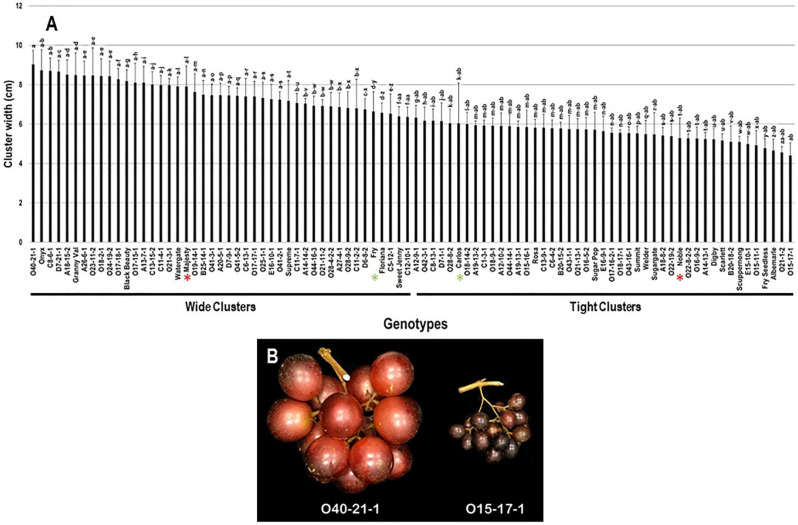
(**A**) Characterization of cluster width (CWI) trait among muscadine population (*n* = 90). The bars represent the mean cluster width (±SD) results from five biological replicates among three years (*n* = 15). The y-axis refers to the cluster width (cm). Means within columns for the same letter followed by different letters differ significantly by Tukey’s test (*p* < 0.05). Based on median cluster width (~6.3 cm), 47.8% and 52.2% of the muscadine population produced wide and tight clusters, respectively. Other details as in Figure 2. (**B**) A representative image of muscadine genotypes exhibiting a maximal (O40-21-1) and a minimal (O15-17-1) cluster width.

The cluster width (CWI) trait demonstrated minor differences among the population with a range of 4.6 cm. The maximal and minimal CWI were recorded in O40-21-1 (9.0 cm ± 0.7) and O15-17-1 (4.4 cm ± 0.6) genotypes. The calculated CWI average among the population was 6.6 cm ± 1.2. Based on the median CWI (~6.3 cm), 47.8% (43 genotypes) and 52.2% (47 genotypes) of the population displayed wide and tight clusters, respectively. The standard commercial table cultivars, Majesty (7.9 cm ± 1.1) and Fry (6.6 cm ± 1.0) were classified among muscadine genotypes showing wide clusters; however, the wine cultivars, Carlos (6.0 cm ± 2.0) and Noble (5.3 cm ± 1.0) belonged to the group representing tight clusters (Figure 3).

#### 2.2.3. Cluster Weight

**Figure 4:**
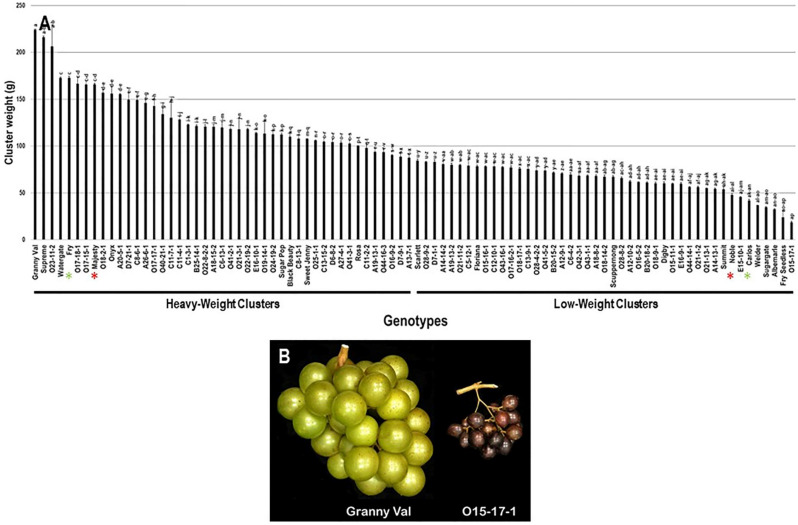
(**A**) Characterization of cluster-weight (CWE) trait among muscadine population (*n* = 90). The bars represent the mean CWE (±SD) results from five biological replicates among three years (*n* = 15). The y-axis refers to the cluster-weight (g). Means within columns for the same letter followed by different letters differ significantly by Tukey’s test (*p* < 0.05). Based on median CWE (~88.1 g), the population was divided into two equal groups of heavy and light clusters. Other details as in Figure 2. (**B**) A representative image of muscadine genotypes exhibiting maximal (Granny Val) and minimal CWE (O15-17-1).

The cluster weight (CWE) trait considerably varied among the population with a wide range of 205.6 g. The maximum CWE was recorded for the Granny Val cultivar (224.0 g ± 0.6), while the lowest CWE was observed in O15-17-1 (18.4 g ± 0.9). The average CWE among the population was estimated at 96.9 g ± 42.7. The median CWE (~88.1 g) divided the population into two equal groups that produced heavy and light clusters weight. As expected, the standard commercial table cultivars, Fry (172.8 g ± 1.5) and Majesty (165.7 g ± 1.1) were classified as members of the muscadine group producing heavy clusters. In contrast, the wine muscadines, Noble (47.8 g ± 1.5) and Carlos (41.9 g ± 1.4) were members of light clusters (Figure 4).

#### 2.2.4. Number of Berries/Cluster

**Figure 5:**
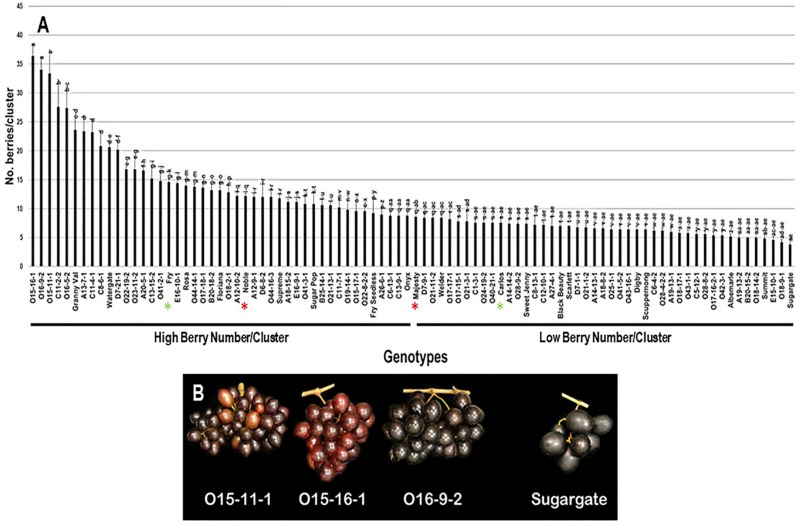
(**A**) Characterization of the number of berries/cluster (N.B/C) trait among muscadine population (*n* = 90). The bars represent the mean N.B/C (±SD) results from five biological replicates among three years (*n* = 15). The y-axis refers to the number of berries/cluster. Means within columns for the same letter followed by different letters differ significantly by Tukey’s test (*p* < 0.05). Based on median N.B/C (~8.7 B/C), the population was divided into two equal groups of high and low number of berries/cluster. Other details as in Figure 2. (**B**) A representative image of the VM genotypes exhibiting maximal N.B/C (O15-11-1, O15-16-1, and O16-9-2) and the muscadine genotype (Sugargate) showing minimal N.B/C.

The number of berries/cluster (N.B/C) trait had a broad range of 32.6 B/C (3.8–36.4 B/C) among the population. The three VM genotypes O15-16-1 (36.4 B/C ± 2.1), O16-9-2 (34.0 B/C ± 2.2), and O15-11-1 (33.4 B/C ± 4.0) displayed the highest N.B/C with insignificant differences between them. However, the muscadine cultivar Sugargate (3.8 B/C ± 0.8) showed the lowest N.B/C. The population had an average of 11.1 B/C ± 6.8. Based on the median N.B/C (~8.7 B/C), the population was separated into two halves of high and low number of berries/cluster. Accordingly, the standard commercial cultivars Fry (14.6 B/C ± 0.5) and Noble (12.2 B/C ± 0.4) were classified among muscadine genotypes exhibiting high N.B/C. However, Majesty (8.6 B/C ± 0.5) and Carlos (7.6 B/C ± 0.9) belonged to the group displaying low N.B/C (Figure 5).

#### 2.2.5. Cluster Compactness

**Figure 6:**
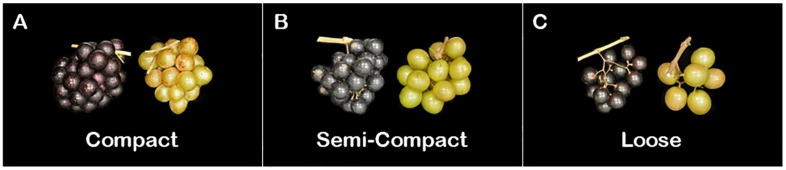
A representative image demonstrates muscadine-grape cluster structure, including (**A**) compact clusters, (**B**) semi-compact clusters, and (**C**) loose clusters.

Cluster compactness (CC) represents the cluster structure or intensity (Figure 6). It was categorized as tightly packed with little to no space in-between berries, termed as compact (Figure 6A). Semi-compact was identified by little air pockets or spaces within the arrangement of the berries in the cluster (Figure 6B). Finally, loose cluster intensity had an open cluster where each berry was easily identifiable and singled out with substantial air pockets around berries (Figure 6C).

**Figure 7:**
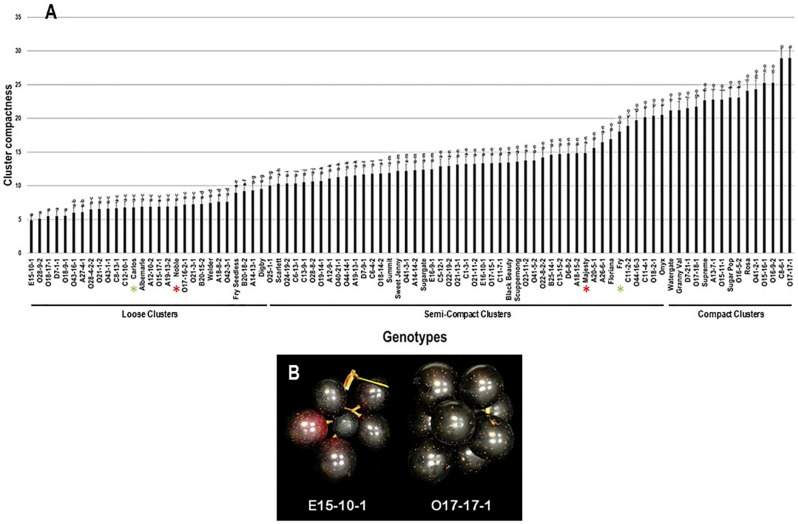
(**A**) Characterization of cluster compactness (CC) trait among muscadine population (*n* = 90). The bars represent the mean CC (±SD) results from five biological replicates among three years (*n* = 15). The y-axis refers to the cluster compactness levels. Means within columns for the same letter followed by different letters differ significantly by Tukey’s test (*p* < 0.05). Based on median CC (~12.2), the population was divided into three groups based on the cluster intensity, loose, semi-compact, and compact. Other details as in Figure 2. (**B**) A representative image of the muscadine genotypes exhibiting minimal (E15-10-1) and maximal (O17-17-1) compactness level.

The average CC was determined visually and calculated using five different clusters. Among the population, the CC trait showed a wide range of 24.1 with minimal and maximal levels of 4.9 and 29.0 for loose and compact clusters, respectively (Figure 7). The average CC among the population was estimated at 13.2 ± 6.1. The population was separated into three main groups based on visual determination and median CC trait (~12.2). The first group, represented by 31.1% of the population (28 genotypes), comprises all genotypes producing loose clusters with CC levels ranged between 4.9 and 10.0. The second group was dominant and represented by 52.2% of the population (47 genotypes). It includes all genotypes producing semi-compact clusters with a CC range of >10.0–20.6. The third group, represented by 16.7% of the population (15 genotypes), consists of all genotypes producing compact clusters with CC range of >20.6 to 29.0. Muscadine genotypes E15-10-1 (4.9 ± 0.7), O28-9-2 (5.1 ± 0.7), O18-17-1 (5.5 ± 0.7), D7-1-1 (5.5 ± 0.7), and O18-9-1 (5.5 ± 0.7) produced the loosest clusters with no substantial differences between them. However, the most compact clusters were recorded for the muscadine genotypes C8-6-1 (28.9 ± 1.5) and O17-17-1 (29.0 ± 1.5). The standard cultivars Carlos (6.8 ± 0.8) and Noble (6.9 ± 0.8) were classified among the muscadine genotypes producing loose clusters. However, Majesty (14.9 ± 1.2) and Fry (18.0 ± 1.3) belonged to the group displaying semi-compact clusters (Figure 7).

#### 2.2.6. Frequency Distribution of Cluster-Related Traits

**Figure 8:**
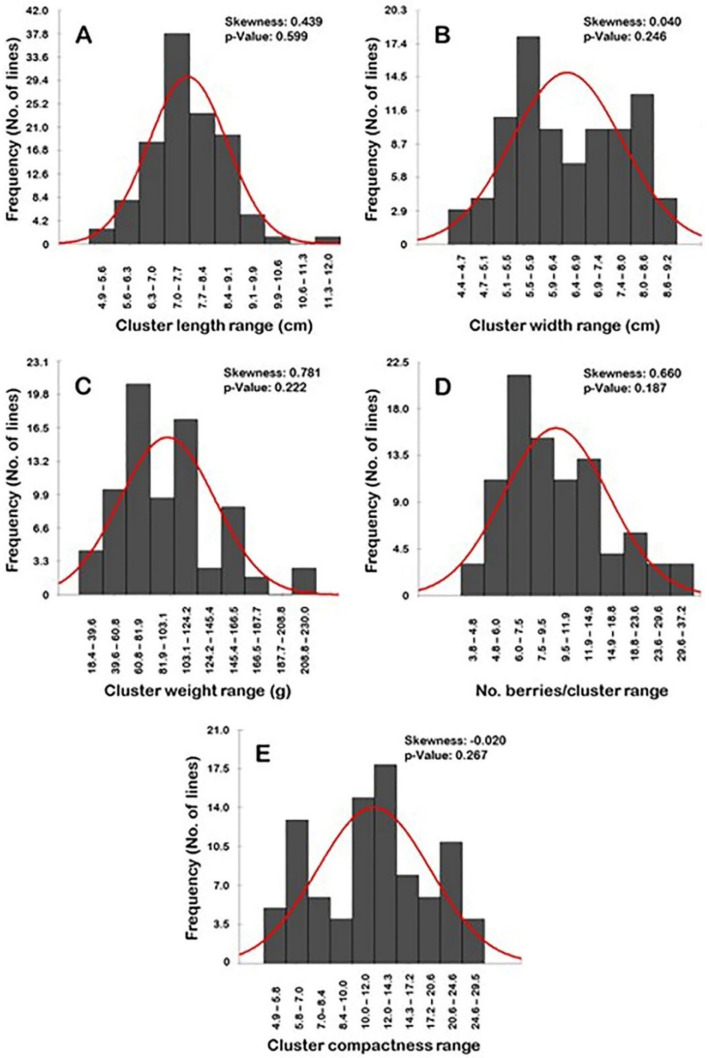
Frequency distribution of the cluster-related traits, including cluster length (**A**), cluster width (**B**), cluster weight (**C**), number of berries/cluster (**D**), and cluster compactness (**E**) of the muscadine population (*n* = 90). The skewness degree and *p*-value of the Kolmogorov-Smirnov normal distribution test are indicated.

Frequency distribution analysis of cluster-related traits suggested a particular distribution pattern for each trait (Figure 8). Interestingly, all of the evaluated characteristics have normal frequency distribution patterns (*p* > 0.05). The distribution behavior for cluster length, width, weight, and the number of berries/clusters were skewed to the right, departing from normality (Figure 8A–D). However, the level of skewness visibly varied among traits, stretching between strong- (i.e., CL, CWE, and N.B/C) to slight-skewed (i.e., CWI). By contrast, the distribution pattern for the cluster compactness (CC) trait was skewed slightly to the left, departing from normality (Figure 8E).

The distribution pattern of the cluster length and width traits has a bimodal distribution (Figure 8A,B). A bimodal distribution usually indicates the existence of two main phenotypes in the population. The two detected phenotypes for the CL trait were designated as short to long clusters (4.9–11.3 cm; 89 genotypes or 98.9% of the population) and very long clusters (>11.3–12.0 cm; 1 genotype or 1.1% of the population). The CL range of 7.0–7.7 cm described the major mode (29 genotypes or ~32.2% of the population) among the population; however, the minor mode was observed within the range of 11.3–12.0 cm (1 genotype or 1.1% of the population). The two phenotypes for CWI were designated as tight (4.4–6.9 cm; 41 genotypes or ~45.6% of the population) and wide clusters (>6.9–9.2 cm; 49 genotypes or ~54.4% of the population). The major CWI mode among the population was observed for the wide clusters with a range of 6.6–6.9 cm (18 genotypes or ~20.0% of the population); however, the minor mode was detected for the wide clusters with a range of 8.0–8.6 cm (13 genotypes or ~14.4% of the population).

The cluster weight trait showed a more complicated pattern since its distribution exhibited a multimodal behavior (Figure 8C). The major mode was described at a low CWE range of 60.8–80.1 g (24 genotypes or ~27.7% of the population). Several minor modes were observed for the higher CWE ranges of 103.1–124.2 g (20 genotypes or ~22.2% of the population), 145.4–166.6 g (10 genotypes or ~11.1% of the population), and 208.8–230.0 g (3 genotypes or ~3.3% of the population). This behavior could be due to the wide variation of cluster weight within the same genotype/vine or obviously among the different genotypes.

Finally, the number of berries/cluster and cluster compactness traits typically have a trimodal distribution. A trimodal distribution usually indicates that three main phenotypes exist in the studied population (Figure 8D–E). In the case of the number of berries/cluster, the three categories were designated as low (3.8–9.5 B/C; 50 genotypes or ~55.6% of the population), intermediate N.B/C (>9.5–18.8 B/C; 28 genotypes or ~31.1% of the population), and a high number of berries/cluster (>18.8–37.2 B/C; 12 genotypes or ~13.3% of the population). The major mode was described at a low N.B/C range of 6.0–7.5 B/C (21 genotypes or ~23.3% of the population). The two minor modes were observed for the intermediate N.B/C range of 11.9–14.9 B/C (13 genotypes or ~14.4% of the population) and the high N.B/C range of 18.8–23.6 B/C (6 genotypes or ~6.7% of the population). In the case of cluster compactness (CC), the three categories were designated as loose clusters (4.9–10; 28 genotypes or ~31.1% of the population), semi-compact clusters (>10.0–20.6; 47 genotypes or ~52.2% of the population), and compact clusters (>20.6–29.5; 15 genotypes or ~16.7% of the population). Interestingly, the identified categories by normal distribution match with the visual compactness characterization, which adds more creditability for the obtained results. The major CC mode was detected for the semi-compact cluster structure with a range of 12.0–14.3 (15 genotypes or ~16.7% of the population). The two minor modes were observed for the loose cluster structure with a range of 5.8–7.0 (13 genotypes or ~14.4% of the population) and the compact cluster structure with a range of 20.6–24.6 (11 genotypes or ~12.2% of the population).

### 2.3. Berry-Related Traits

#### 2.3.1. Berry Length

**Figure 9:**
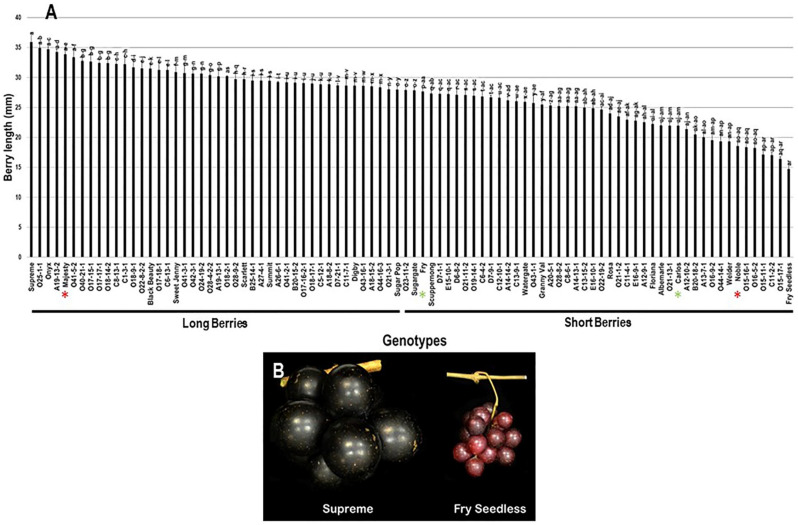
(**A**) Characterization of berry length (BL) trait among muscadine population (*n* = 90). The bars represent the mean BL (±SD) results from five biological replicates among three years (*n* = 15). The y-axis refers to the berry length (mm). Means within columns for the same letter followed by different letters differ significantly by Tukey’s test (*p* < 0.05). Based on median BL (~27.9 mm), the population was divided into two groups of long berries (44 genotypes, 48.9% of the population) and short berries (46 genotypes, 51.1% of the population). Other details are as in Figure 2. (**B**) A representative image of the muscadine genotypes exhibiting maximal (Supreme) and minimal (Fry Seedless) berry length.

The population displayed a wide range of berry length (BL) estimated at 21.2 mm, where the muscadine cultivars Supreme (35.9 mm ± 2.0) and Fry Seedless (14.7 mm ± 1.0) exhibited the highest and lowest recorded BL, respectively. The average BL among the population was estimated at 27.0 mm ± 4.7. The median BL (~27.9 mm) divided the population into two groups, where 48.9% (44 genotypes) and 51.1% (46 genotypes) of the population displayed long and short berries, respectively. Accordingly, the standard cultivar Majesty (33.9 mm ± 1.1) was classified among the group producing long berries. However, Fry (27.6 mm ± 0.6), Carlos (21.9 mm ± 0.6), and Noble (18.6 mm ± 0.7) were members of the group producing short berries (Figure 9).

#### 2.3.2. Berry Width

**Figure 10:**
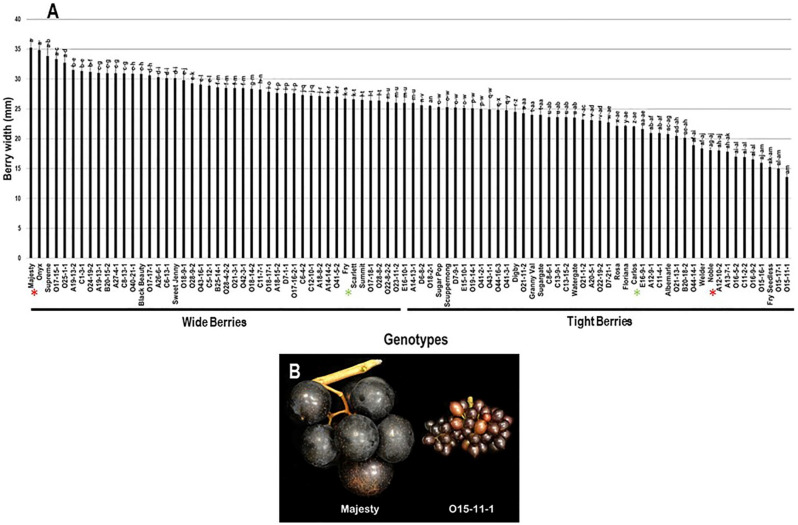
(**A**) Characterization of berry width (BWI) trait among muscadine population (*n* = 90). The bars represent the mean BWI (±SD) results from five biological replicates among three years (*n* = 15). The y-axis refers to the berry width (mm). Means within columns for the same letter followed by different letters differ significantly by Tukey’s test (*p* < 0.05). Based on median BWI (~26.0 mm), the population was divided into two equal groups of wide and tight berries. Other details are as in Figure 2. (**B**) A representative image of the muscadine genotypes exhibiting maximal (Majesty) and minimal (O15-11-1) berry width.

The berry width (BWI) trait revealed a substantial difference among the population with a range of 21.7 mm. The highest BWI was observed in Majesty (35.3 mm ± 1.5) and Onyx (34.8 mm ± 1.6) with no significant difference between them; however, the VM genotype O15-11-1 presented the lowest BWI (13.6 mm ± 0.5). The population had an average BWI of 25.5 mm ± 4.8. Based on the median BWI (~26.0 mm), the population was separated into two equal groups, producing wide and tight berries. As expected, the standard table cultivars, Majesty and Fry (26.7 mm ± 1.0), were classified as members of the muscadine group producing wide berries. In contrast, the wine muscadines, Carlos (22.0 mm ± 0.1) and Noble (18.1 mm ± 0.4) were members of tight berries group (Figure 10).

#### 2.3.3. Berry Weight

**Figure 11:**
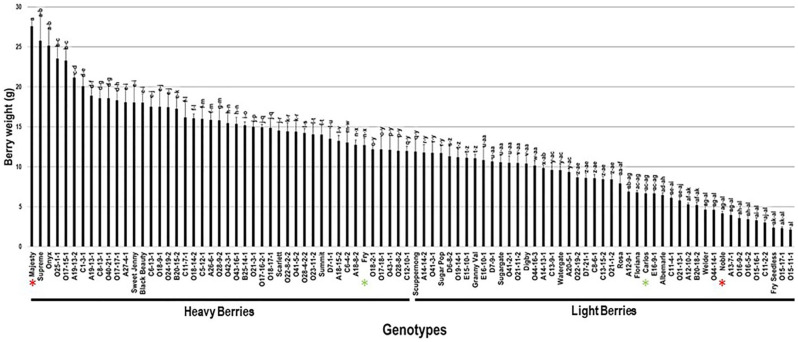
Characterization of berry weight (BWE) trait among muscadine population (*n* = 90). The bars represent the mean BWE (±SD) results from five biological replicates among three years (*n* = 15). The y-axis refers to the berry weight (g). Means within columns for the same letter followed by different letters differ significantly by Tukey’s test (*p* < 0.05). Based on median BWE (~11.9 g), the population was divided into two equal groups of heavy and light berries. Other details are as in Figure 2. A representative image for the muscadine genotypes exhibiting maximal and minimal berry weight is as in Figure 10.

The berry weight trait (BWE) showed significant differences among the population with an estimated range of 25.4 g. Majesty (27.6 g ± 0.8), Supreme (25.8 g ± 3.5), and Onyx (25.2 g ± 2.2) cultivars produced the heaviest berries. On the other side, the Fry Seedless, O15-17-1, and the VM O15-11-1 displayed the lowest berry weight estimated at 2.4 g ± 0.4, 2.3 g ± 0.1, and 2.1 g ± 0.2, respectively. The average BWE among the population was estimated at 12.2 g ± 5.6. The BWE median (~11.9 g) split the population into two equal groups that produced heavy and lightweight berries. The standard table cultivars, Majesty and Fry (12.7 g ± 0.9), were classified as members of the muscadine group producing heavy berries. However, the wine muscadines, Carlos (6.7 g ± 0.4) and Noble (4.2 g ± 0.3), were members of the light berries group (Figure 11).

#### 2.3.4. Number of Seeds/Berry (N.S/B)

**Figure 12:**
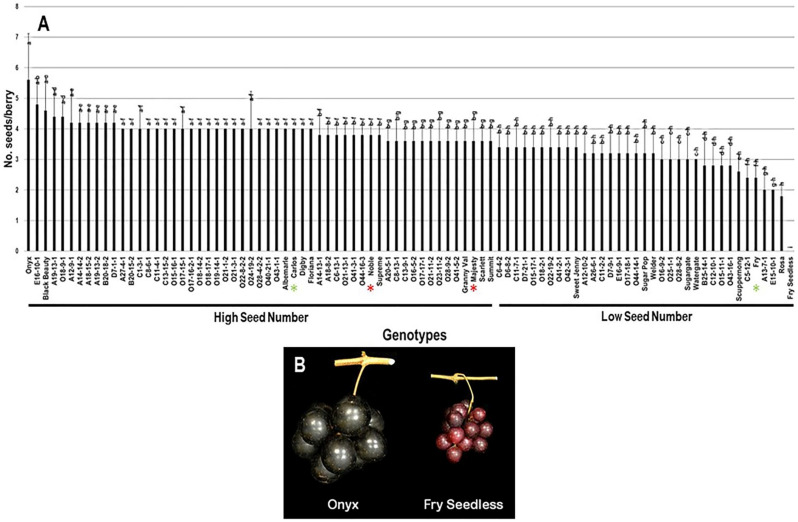
(**A**) Characterization of number of seeds/berry (N.S/B) trait among muscadine population (*n* = 90). The bars represent the mean N.S/B (±SD) results from five biological replicates among three years (*n* = 15). The y-axis refers to the number of seeds/berry. Means within columns for the same letter followed by different letters differ significantly by Tukey’s test (*p* < 0.05). Based on median N.S/B (~3.6), the population was divided into two equal groups of a high and low seed number. Other details are as in Figure 2. (**B**) A representative image of the muscadine genotypes exhibiting maximal (Onyx) and minimal (Fry Seedless) number of seeds/berry.

The average number of seeds/berry (N.S/B) was 3.6 seeds ± 0.7. The N.S/B attribute had a minor range of 5.6 among the population. The muscadine cultivar Onyx had the most seed events present in berries, totaling 5.6 S/B ± 1.5. At the same time, Rosa muscadine cultivar had the least recorded N.S/B of 1.8 S/B ± 0.4. The previous results are accurate among seeded muscadines since the muscadine genotype Fry Seedless had the lowest seed number statistically. Fry Seedless genotype exhibits a parthenocarpy fruit-set program with no seed events developed during growth. It is tempting to highlight that Fry Seedless was the only seedless genotype in the characterized population. The median N.S/B (~3.6 S/B) divided the population into two high and low seed number/berry groups. High seed numbers accounted for 55 genotypes (61.1% of the population), while low seed numbers grossed 35 genotypes (38.9% of the population). Accordingly, the muscadine cultivars Carlos (4.0 S/B ± 0), Noble (3.8 S/B ± 0.4), and Majesty (3.6 S/B ± 0.9) were classified among the muscadine genotypes displaying high seeds number/berry. However, Fry (2.4 S/B ± 0.5) belonged to the group showing low seed number/berry (Figure 12).

#### 2.3.5. Weight of Seeds/Berry (W.S/B)

**Figure 13:**
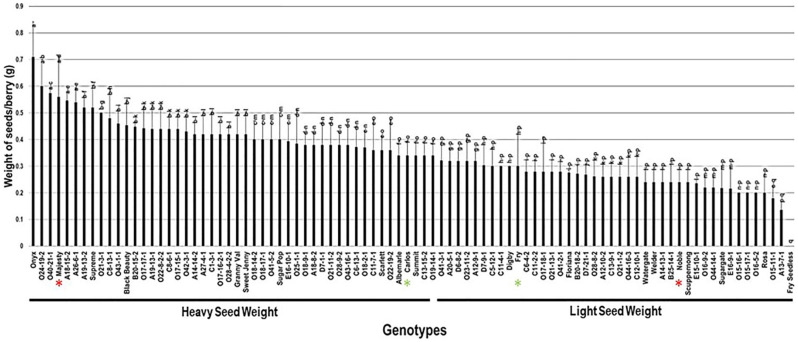
Characterization of the weight of seeds/berry (W.S/B) trait among muscadine population (*n* = 90). The bars represent the mean W.S/B (±SD) results from five biological replicates among three years (*n* = 15). The y-axis refers to the weight of seeds/berry (g). Means within columns for the same letter followed by different letters differ significantly by Tukey’s test (*p* < 0.05). Based on median W.S/B (~0.34 g), the population was divided into two equal groups of heavy and light seed weight. Other details are as in Figure 2. A representative image of the muscadine genotypes exhibiting maximal and minimal weight of seeds/berry is as in Figure 12.

The weight of seeds/berry (W.S/B) had a wide range among the population, estimated at 0.71 g. The W.S/B character was, to some extent, reflective to the number of seeds as the muscadine cultivar Onyx displayed the maximum W.S/B of 0.71 g ± 0.13. In comparison, the A13-7-1 muscadine genotype had the least recorded W.S/B of 0.14 g ± 0.03. However, the muscadine genotype Fry Seedless still statistically displayed the lowest weight of seeds/berry due to the parthenocarpy fruit-set, as described previously. The W.S/B trait had a mean of 0.35 g ± 0.11. The median of W.S/B (~0.34 g) divided the population into two groups. Among them, 53% (48 genotypes) produced heavy seed weight, and 47% (42 genotypes) formed light seed weight. The previous analysis suggested that muscadine cultivars Majesty (0.56 g ± 0.15) and Carlos (0.34 g ± 0.05) exhibited a heavy seed weight character. However, Fry (0.30 g ± 0.14) and Noble (0.24 g ± 0.05) belonged to the group displaying light seed weight/berry (Figure 13).

#### 2.3.6. Berry Firmness

**Figure 14:**
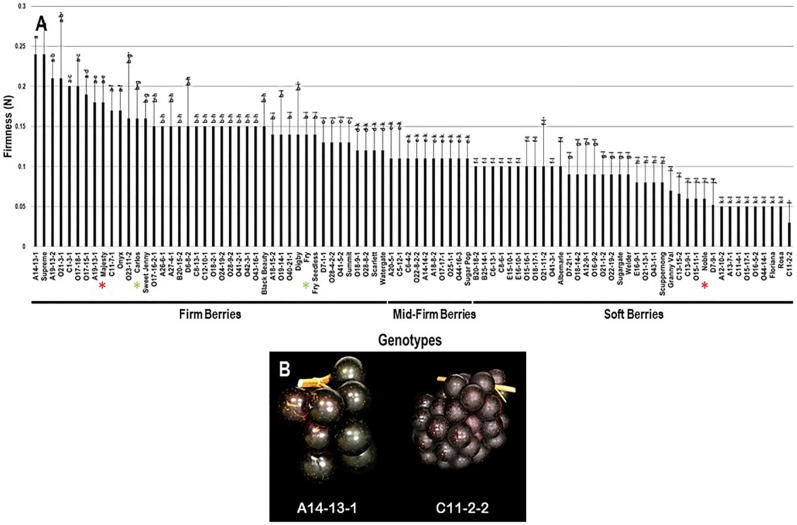
(**A**) Characterization of firmness (FF) trait among muscadine population (*n* = 90). The bars represent the mean FF (±SD) results from five biological replicates among three years (*n* = 15). The y-axis refers to the firmness (N). Means within columns for the same letter followed by different letters differ significantly by Tukey’s test (*p* < 0.05). The population was divided into a firm, mid-firm, and soft berries based on median FF (~0.11 N). Other details are as in Figure 2. (**B**) A representative image of the muscadine genotypes exhibiting maximal (A14-13-1) and minimal (C11-2-2) berry firmness.

The firmness trait (FF) had an extensive range among the population estimated at 0.21 N. The highest berry firmness was detected in two muscadine genotypes, the A14-13-1 (0.24 N ± 0.02) and Supreme (0.24 N ± 0.04). The lowest FF value was identified in the C11-2-2 muscadine (0.03 N ± 0.02). The FF had an average value of 0.12 N ± 0.05 among the population. The median FF (~0.11 N) divided the population into three groups, designated as firm, mid-firm, and soft berries (Figure 14). Firm berries (0.12–0.24 N) accounted for 46.7% of the population (42 genotypes). However, mid-firm berries (0.11 N) were represented by 11.1% of the population (10 genotypes), and soft berries (0–0.10 N) were expressed by 42.2% of the population (38 genotypes). Accordingly, muscadine cultivars Majesty (0.18 N ± 0.03), Carlos (0.16 N ± 0.04), and Fry (0.14 N ± 0.02) were classified among firm berries. However, the Noble (0.06 N ± 0.02) muscadine cultivar belonged to the group displaying soft berries (Figure 14).

#### 2.3.7. Scar Pattern (SP)

**Figure 15:**
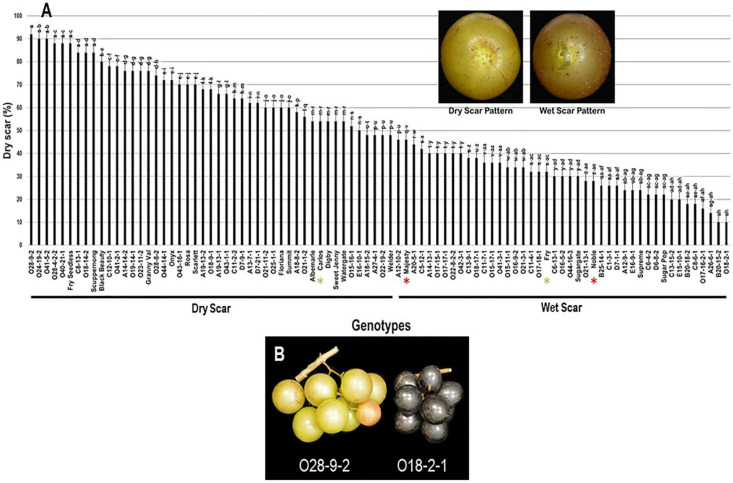
(**A**) Characterization of scar pattern (SP) trait among muscadine population (*n* = 90). The bars represent the mean SP (±SD) results from five biological replicates among three years (*n* = 15). The y-axis refers to the dry scar pattern (%). Means within columns for the same letter followed by different letters differ significantly by Tukey’s test (*p* < 0.05). Based on median SP (~48%), the population was divided into two equal groups of dry scar and wet scar. Other details are as in Figure 2. A close-up view of muscadine berries showing dry scar vs. wet scar patterns in muscadine berries is indicated. (**B**) A representative image of the muscadine genotypes exhibiting extreme dry (O28-9-2) and wet (O18-2-1) berry scar.

The scar pattern trait (SP) visibly varied among the population and displayed a wide range of 82%. Muscadine genotypes O28-9-2 (92% ± 1.8), O24-19-2 (90% ± 2.0), and O41-5-2 (90% ± 1.5) showed the highest dry scar pattern. In contrast, both B20-15-2 (10% ± 0.6) and O18-2-1 (10% ± 0.8) muscadines had the lowest dry scar (majority wet scar). The population had an average of 49.7% ± 22.4 dry scar. The median SP trait (~48%) separated the population into two groups of high and low (wet) dry scar patterns. The high dry scar pattern (48–92%) accounted for 47 genotypes (52.2% of the population). However, the low dry scar pattern (10–46%) accounted for 43 genotypes (47.8% of the population). The muscadine cultivar Carlos (54% ± 1.1) was classified among muscadine genotypes displaying dry scar pattern. However, Majesty (46% ± 1.9), Fry (32% ± 0.6), and Noble (28% ± 1.1) belonged to the group showing wet scar pattern (Figure 15).

### 2.4. Frequency Distribution of Berry-Related Traits

**Figure 16:**
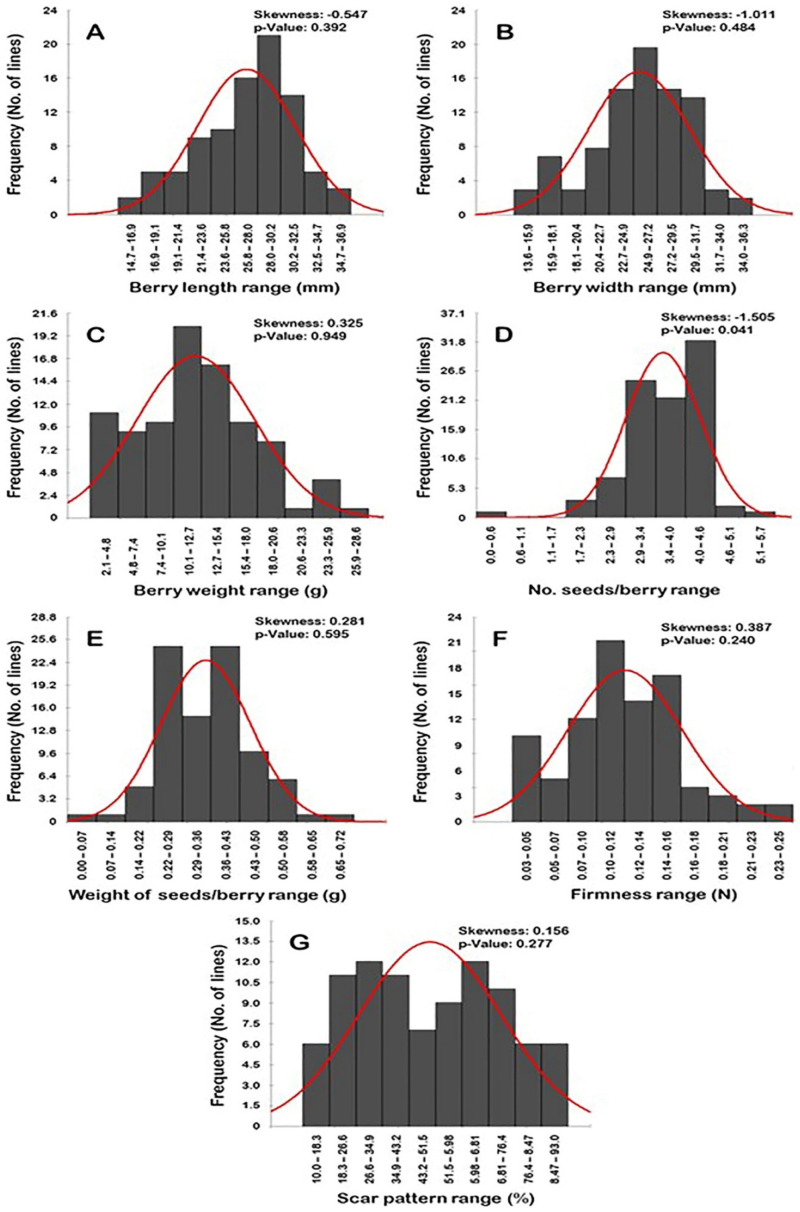
Frequency distribution of the berry-related traits, including berry length (**A**), berry width (**B**), berry weight (**C**), number of seeds/berry (**D**), the weight of seeds/berry (**E**), firmness (**F**), and scar pattern (**G**) of the muscadine population (*n* = 90). The skewness degree and *p*-value of the Kolmogorov-Smirnov normal distribution test are indicated.

Frequency distribution analysis of berry-related traits suggested a distinguishable distribution pattern among traits (Figure 16). Interestingly, all of the evaluated traits exhibited normal frequency distribution patterns, excluding the number of seeds/berry (N.S/B) trait (*p* > 0.05). The distribution behavior for berry length (BL), width (BWI), and N.S/B traits was skewed largely to the left, departing from normality (Figure 16A,B,D). By contrast, the distribution pattern for berry weight (BWE), weight of seeds/berry (W.S/B), firmness (FF), and dry scar pattern (SP) characters were skewed to the right, departing from normality (Figure 16C,E–G).

The BL trait typically exhibited a unimodal distribution pattern, by which the BL range of 28.0–30.2 mm represented the major mode (21 genotypes or ~23.3% of the population) (Figure 16A). This pattern of distribution suggests that berry length is likely regulated quantitatively in the studied population.

The distribution pattern of the BWI, W.S/B, and SP traits exhibited a bimodal distribution behavior (Figure 16B,E,G). A bimodal distribution suggested the existence of two main phenotypes in the population. The BWI trait was separated as tight (13.6–20.4 mm; 13 genotypes or ~14.4% of the population) and wide berry (>20.4–36.3 mm; 77 genotypes or ~85.6% of the population). The major BWI mode among the population was observed for the wide BWI with a range of 24.9–27.2 mm (20 genotypes or ~22.2% of the population); however, the minor mode was detected for the tight BWI range of 15.9–18.1 mm (7 genotypes or ~7.8% of the population). The W.S/B was manifested as light seed weight (0–0.36 g; 47 genotypes or ~52.2% of the population) and heavy seed weight (>0.36–0.72 g; 43 genotypes or ~47.8% of the population). The W.S/B exhibited a particular mode among the population due to the detection of two dominant major modes. The two modes were observed for the light W.S/B with a range of 0.29–0.36 g and the heavy W.S/B with a range of 0.43–0.50 g. Each of these two modes was represented 25 genotypes or ~27.8% of the population. Finally, the SP character was established as a low dry scar or a wet scar (10–51.5%; 47 genotypes or ~52.2% of the population) and dry scar (>51.5–93%; 43 genotypes or ~47.8% of the population). As in the W.S/B trait, the population exhibited two major SP modes. The two modes were described as a modest wet scar pattern (26.6–34.9%) and a modest dry scar pattern (59.8–68.1%). Each of these two modes was represented by 12 genotypes or ~13.3% of the population.

The BWE, N.S/B, and FF traits typically have a trimodal distribution in the studied population. A trimodal distribution usually suggested three main phenotypes in the population (Figure 16C,D,F). The BWE trait was split into three groups designated as low (2.1–7.4 g; 20 genotypes or ~22.2% of the population), medium (>7.4–20.6 g; 64 genotypes or ~71.1% of the population), and heavy berry weight (>20.6–28.6 g; 6 genotypes or ~6.7% of the population). The major BWE mode was described at the modest BWE range of 10.1–12.7 g (20 genotypes or ~22.2% of the population). The two minor modes were observed for the low BWE range of 2.1–4.8 g (11 genotypes or ~12.2% of the population) and the heavy BWE range of 23.3–25.9 g (4 genotypes or ~4.4% of the population). In the N.S/B trait, the three categories were established as seedless (0–0.57; 1 genotype or ~1.1% of the population), intermediate (1.7–4.0; 55 genotypes or ~61.1% of the population), and high seed number (>4.0–5.7; 34 genotypes or ~37.8% of the population). The major mode was detected for the high N.S/B with a range of 4.0–4.6 S/B (31 genotypes or ~34.4% of the population). The two minor modes were observed for the modest N.S/B with a range of 2.9–3.4 S/B (24 genotypes or ~26.7% of the population) and the low N.S/B with a range 0–0.57 S/B (1 genotype or ~1.1% of the population). Finally, the FF trait was manifested as soft (0.03–0.07 N; 15 genotypes or ~16.7% of the population), mid-firm (>0.07–0.14; 47 genotypes or ~52.2% of the population), and firm (>0.14–0.25; 28 genotypes or ~31.1% of the population). The major FF mode was described at the mid-firm berries with a range of 0.10–0.12 N (21 genotypes or ~23.3% of the population). The two minor modes were observed for the firm berries with a range of 0.14–0.16 N (17 genotypes or ~18.9% of the population) and for the soft berries with a range of 0.03–0.05 N (10 genotypes or ~11.1% of the population).

### 2.5. Classification of Muscadine Genotypes Based on the Evaluated Traits

**Figure 17:**
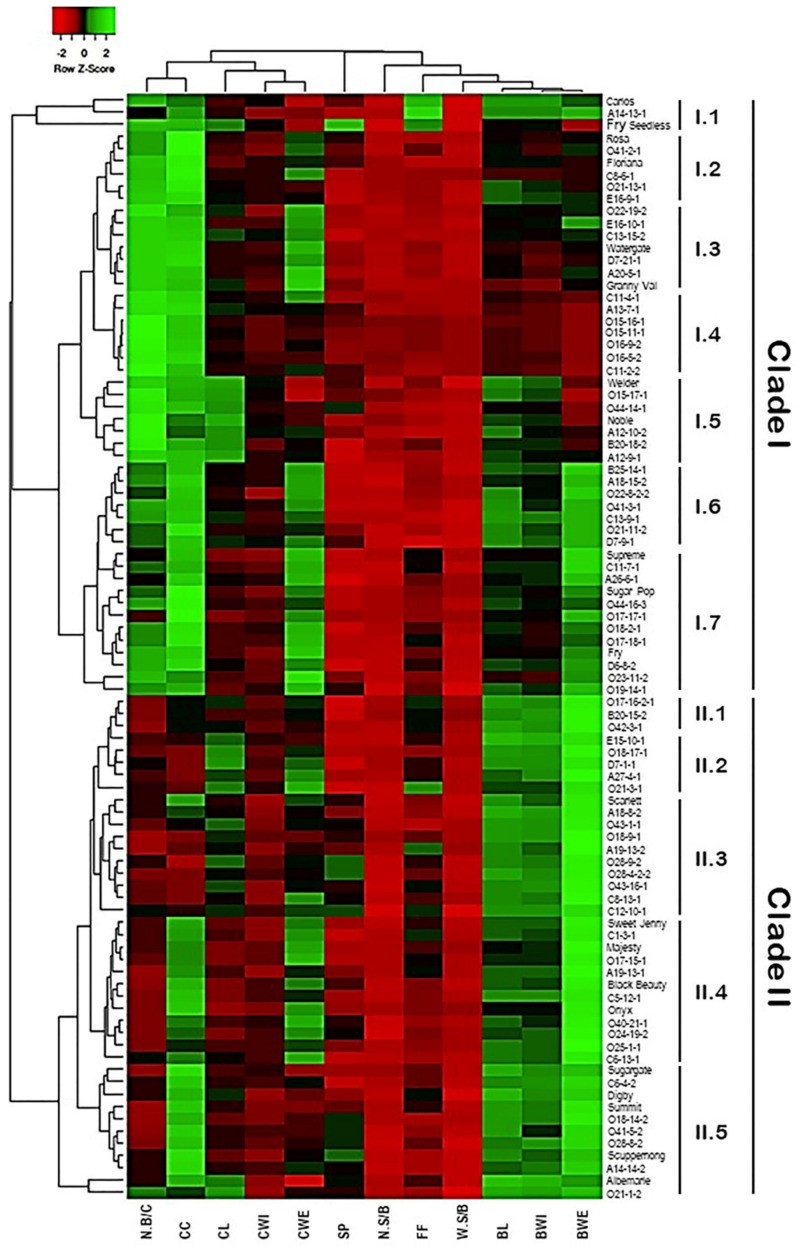
Hierarchical clustering of the different evaluated traits of muscadine population (*n* = 90). Data of different traits, including variables of cluster length (CL), cluster width (CWI), cluster weight (CWE), number of berries/cluster (N.B/C), cluster compactness (CC), berry length (BL), berry width (BWI), berry weight (BWE), number of seeds/berry (N.S/B), the weight of seeds/berry (W.S/B), firmness (FF), and scar pattern (SP) are presented as an average of five biological replicates among three years (*n* = 15). The log2-transformed values of each character are represented by colors. Green and red boxes indicate higher and lower values, respectively. The color change is proportional to the two extremes (see the color scale at the top of the figure).

A hierarchical cluster map was constructed to classify the muscadine population based on their diverse cluster and berry physical traits (Figure 17). Despite the variation among individuals for the different traits, the hierarchical clustering separated the population into two main groups. The first group represented by the top clade comprises all genotypes exhibiting high N.B/C and CC (49 genotypes or ~54.4% of the population). This clade was further separated into seven subclades based on the other characteristics. The most remarkable subclades were I.1, I.3, and I.5. In addition to the main two characters of the group, the I.1, I.3, and I.5 subclades showed enhanced FF, CWE, and CL characteristics, respectively. The subclades I.6 and I.7 shared similar improved characteristics of CWE and BWE traits; however, subclade I.6 exhibited an extra feature of enriched BL. By contrast, the second group represented by the bottom clade includes all genotypes displaying accelerated berry characteristics, including BL, BWI, and BWE. The clade II was further separated into five subclades based on the other characteristics. The most remarkable subclades were II.4 and II.5 that shared the character of high CC levels; however, subclade II.4 demonstrated an extra feature of enriched BWE character.

### 2.6. Principal Component Analysis of Different Evaluated Traits

**Figure 18:**
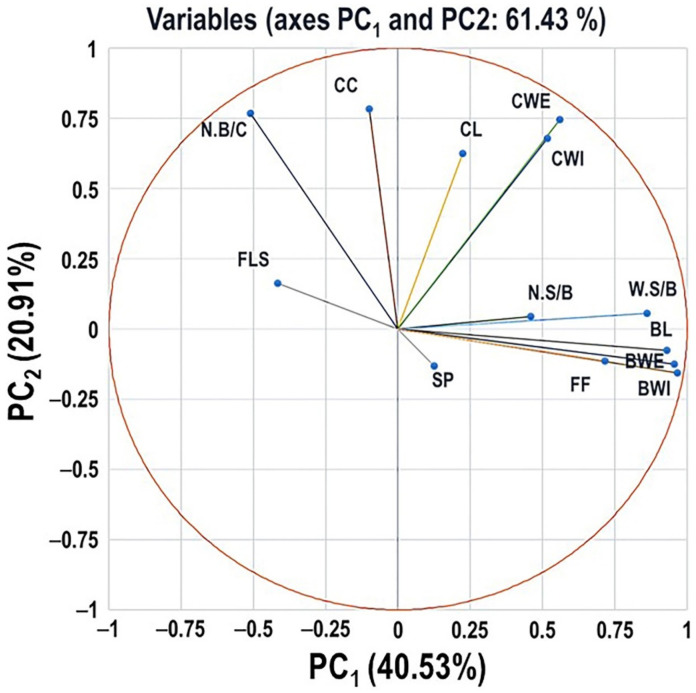
Principal Component Analysis (PCA) scatter plots of the different cluster- and berry-related traits, including variables of cluster length (CL), cluster width (CWI), cluster weight (CWE), number of berries/cluster (N.B/C), cluster compactness (CC), berry length (BL), berry width (BWI), berry weight (BWE), number of seeds/berry (N.S/B), the weight of seeds/berry (W.S/B), firmness (FF), and scar pattern (SP). The flower structure (FLS) trait was also covered in the analysis. The scatter was generated using the average of three biological replicates. According to the PCA model, 40.53% and 20.91% of the variance were explained by the first principle component (PC_1_) and the second principle component (PC_2_), respectively.

Principle components analysis (PCA) was performed using the data from the assessment of 90 muscadine genotypes to identify groups of traits coordinating cluster and berry physical characteristics (Figure 18). However, to understand better these interactions, we included the flower structure component—FLS (female or hermaphroditic) in the analysis. Application of PCA varimax rotation has permitted identifying two components that explained 40.53 and 20.91%, respectively, with 62.13% cumulative eigenvalues of data variance. Eigenvalues of the third and fourth PCs were negligible (51.99% and 48.68%), and thereby they are not discussed further. The PC_1_ showed the strongest positive correlations with BL, BWI, BWE, FF, and W.S/B. A less significant positive correlation was observed with N.S/B. Despite that the SP trait belongs to the same group, it exhibited a non-significant correlation with PC_1_. A modest significant negative correlation was detected between FLS and PC_1_. The PC_2_ was strongly positively correlated with CL and CC. However, a less significant positive correlation was observed with CWI, CWE, and N.B/C.

### 2.7. Dissimilarity Matrix Analysis among the Population

**Figure 19:**
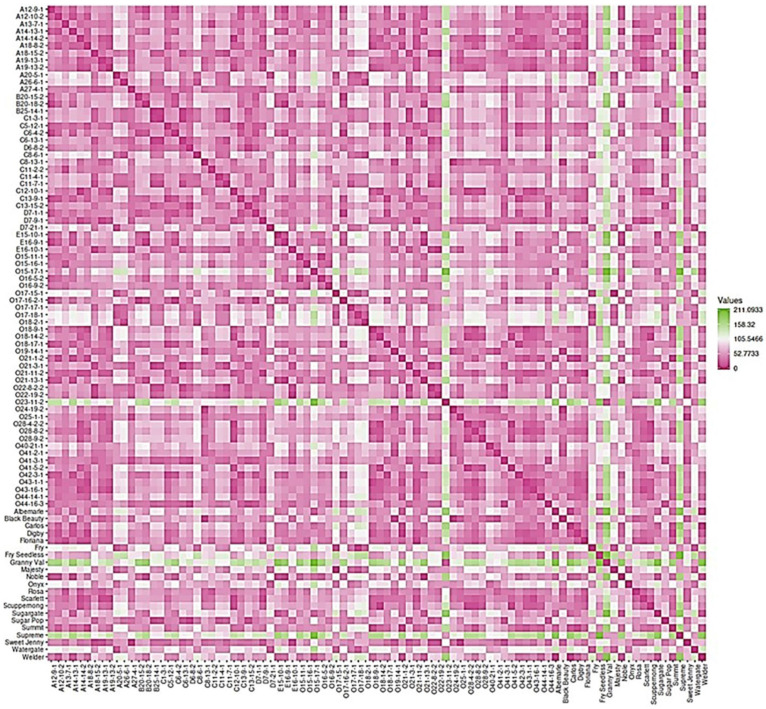
Dissimilarity matrix showing the distances among the genotypes. The gradient of color indicates the distance between genotypes; the green color denotes the highest dissimilarity, and the pink color means the lowest genetic distance. In addition, the pink color represents the diagonal.

The 90 genotypes analyzed in this study showed a high level of diversity with little population structure. The dissimilatory matrix revealed an overall low level of relatedness with very few pairs of closely related genotypes within the population (Figure 19). Despite the distinct background of muscadines and VMs, the VM genotypes were not distinguishable from muscadines. Interestingly, some muscadine genotypes were noticeably divergent from the population, including O23-11-2, Granny Val, and Supreme. Data analysis revealed the enhanced physical cluster and berry characteristics of the highlighted genotypes.

## 3. Discussion

Biometrics is the measurement of unique physical quality traits. Cluster morphology is one of the key parameters that influence cluster health status (cluster architecture and compactness), size characteristics, and yield [16]. Evaluating cluster architecture, the overall shape of a cluster and what defines “good” architecture is important to both table and wine grape breeders and growers [17]. For table grapes, large and loose clusters with definitive shoulders and large berries that attract consumers are key visible feature. In wine grapes, reducing cluster compactness and small berry size are the priorities. Highly compact clusters are more likely to develop sour rot or *Botrytis* bunch rot, and small berries ensure a high skin to flesh ratio—desirable particularly for extraction of flavors and color in red wine varieties [17]. These quality characteristics are acknowledged to some extent in bunch grapes. However, in muscadine grapes these characteristics are less important, particularly for some traits as cluster shape. The main reason is the nature of the muscadine cluster, which is characterized by woody rachis and non-symmetric shape.

In this study, genotypes showed vast differences in cluster-related traits, including cluster weight (CWE), number of berries/cluster (N.B/C), and compactness level (CC). However, the cluster length (CL) and width (CWI) attributes did not considerably vary among population. Cluster compactness can be affected by several traits, including berry number and size, rachis branching, length, and even rachis angle. Evaluating each of these traits is time-consuming and a challenge for grape breeding programs [17]. Assessment of muscadine cluster’s architecture and morphology requires the deconstruction of the cluster to retrieve the maximum data for the quality index. In this study, the CWE trait showed a more complicated pattern since its distribution exhibited a multimodal behavior. This pattern could be due to the wide variation of cluster weight within a muscadine genotype or obviously between the different genotypes or even due to the severe rain during the season, resulting in the loss of immature and mature berries.

Muscadine berries have distinct physical characteristics from bunch grapes in terms of large berry size, thick skin, large seeds, and type of fresh consumption [18]. Most often, muscadines are eaten fresh or used for producing wine and preserves. Due to the large berry size, poor cluster shape, and the woody form of the rachis, the muscadine grape is sold freshly in the form of separated berries rather than entire clusters. Accordingly, the visual attributes of muscadine berries and their physical/mechanical properties are critical factors in the consumer acceptability and sensory perception [19]. For fresh consumption, mechanical properties are among the most important factors determining the eating quality of table grapes. Sensory attributes, such as skin friability and thickness, and flesh firmness, have been proposed to characterize commercial table grape cultivars [2,20]. Many of these sensory descriptors can be evaluated by instrumental measurements, and in many instances, high correlations with the mechanical parameters determined by texture analysis were observed [21,22]. The major challenge for the grape and wine industry is to replace the time-consuming laboratory analyses used in quality monitoring with new techniques that are fast, precise, and accurate. Due to the difficulties associated with sensory evaluation, there is a need for simple, reliable, and objective techniques to assess fruit qualities.

Berry size is widely recognized as an essential factor determining wine grape quality. However, this concept has gained acceptance based on intuition and implicit assumptions with little experimental evidence [23]. Singleton [24] estimated the role of berry size by removing and adding juice at crushing. However, no measurements were conducted to indicate that the juice/solids were greater in larger berries. In its most simplistic form, the relationship in which larger berries are less desirable is described because of water diluting solutes of importance for wine flavor [23]. This explanation lacks important physiological factors of berry size. The grape berries do not grow by ‘pumping’ water into a vessel (berry) of flavor solutes. Berries attain size via a double sigmoid growth habit [25,26].

Firmness is one of the leading indicators in judging the organoleptic quality of grapes for fresh consumption, a desirable feature for good storage, shelf life, and eating quality. Consumers prefer grapes with firmer flesh above those with soft flesh. Firmer berries are commonly accepted to have better-eating quality more freshness, and extended shelf life capacity [27]. The loss of firmness in grapes occurs due to the loss of water or changes in the cell-wall structure, resulting from inadequate storage conditions or extended time between harvest and marketing [28]. Firm, crunchy berries are considered a sign of freshness and health while allowing longer storage time and permitting the fruit to reach market in optimal condition [29]. In this study, the population was divided into three groups: a firm, mid-firm, and soft berries. Therefore, firmness may influence the texture desirability of grapes, and, in those varieties with thick skins, if not associated with a high skin friability; it would limit their commercial acceptance [2]. On the other hand, the thickness and toughness of the skin contribute to the resistance of the grape to fungal pathogens and handling injury during harvest, packing, transport, and storage [30].

Berries should be firm with a dry picking scar so that leaked juice does not promote rotting and stickiness. Fresh-market muscadines require flavorful, disease-free, large-fruited grape with a dry picking scar or a wet scar that can dry quickly, prolonging their shelf life [31]. A stem scar is the primary entry point for decay; therefore, it is essential that the scar does not tear during harvest [32]. The scar pattern, described as wet or dry, was evaluated via characterizing the behavior on a 50-berry basis. In nature, some muscadine berries often abscise from the cluster (shatter) at maturity. Unfortunately, many cultivars tend to have stem scars that remain open in the center or the skin tears around the scar. This pattern is known as having a wet scar, which is deleterious as it provides an entrance into the berry for mold, and the juice can leak from the scar and get the berries sticky. For the fresh market, it is crucial to minimize the wet scars as possible. No cultivar is perfect, but some cultivars are performing better for producing dry scar berries. Such cultivars are preferred for fresh markets, especially where berries will be stored to maintain their qualities before the sale (better shelf life).

PCA plot divided different traits into four main groups. The first group comprises all berry-related traits. The second group covers cluster-related traits coordinating cluster size (CL, CWI, and CWE); however, the third group includes cluster-related traits synchronizing cluster compactness (N.B/C and CC). Finally, the fourth group contains the flower structure (FLS) trait. The group of cluster size displayed a significant positive correlation with the berry-related group and the cluster compactness group; however, no apparent correlation was detected between groups 1 and 3. In the meantime, the FLS trait has a significant positive correlation with the cluster compactness group and contradictory contribution to berry-related traits. Based on the previous analysis, it is tempted to speculate that hermaphroditic flower structure is associated with compact clusters exhibiting small berries in terms of size and weight. In other mean hermaphroditic flower structure is associated with muscadine genotypes suitable for wine production. At the same time, the vice-versa relation is accurate, as the female flower structure is associated with clusters exhibiting large berries in terms of size and weight or muscadine genotypes suitable for fresh consumption. Based on the location of the SP variable in the scatter, it seemed to have a minor contribution to the total variance. In addition, seeds are the primary source of nutrients and hormones necessary to coordinate berry development and growth. Accordingly, the number and weight of seeds present inside the berry play crucial roles in berry growth, maturation, and different quality attributes [33]. Despite this fact, it is difficult to explain the quantitative relationship between the seed number and the size or quality of the berry, even in a given cultivar. The previous results suggested that, as expected, enhanced seed characters in terms of number and weight were associated with large berry size and heavy berry weight.

## 4. Materials and Methods

### 4.1. Plant Material

The muscadine population used in this study was generated as part of the grape breeding program at the Florida Agricultural and Mechanical University’s (FAMU) and Center for Viticulture and Small Fruit Research (CVSFR). For this study, 90 muscadine genotypes, including 21 standard cultivars, 60 breeding lines, and 9 *Vitis x Muscadinia* hybrids (VM hybrids), were selected based on vines age with at least 5-years old vines to ensure stable productivity. A complete list of used genotypes and their flower structure is presented (Table S1). The muscadine grape berries from each genotype were collected at the time of optimum harvest maturity, as determined by berry softness and color. All evaluated parameters were measured for three consecutive years. For all evaluated traits the well-characterized muscadine commercial cultivars Carlos, Noble, Fry, and Majesty were used as controls [34,35,36,37].

### 4.2. Cluster-Related Traits

The cluster-related traits were represented by a set of sub-traits, including measurable and calculated traits (Figure S1). Cluster-length (CL) was assessed as the distance between the apical tip and the highest point on the end of each cluster, including the peduncle, rachis, pedicel, and berries. Cluster-width (CWI) was measured as the distance between the two extreme points of the equatorial zone of each cluster. Cluster weight (CWE) was measured using a VWR 3001E-series digital scale and recorded in grams (VWR International, Radnor, PA, USA). The berries were gently removed from each cluster and counted to count the number of berries/cluster (N.B/C). The level of cluster compactness (CC) was measured following the next equation:

Level of Cluster Compactness (CC) = [No. of berries/cluster × Berry size (mm)]/[Rachis length (cm) × Pedicel Length (mm)]

For this formula, the berry size variable was expressed by calculating the average between berry length (mm) and berry width (mm).

### 4.3. Berry-Related Traits

The berry-related traits were expressed using a set of sub-traits, including measurable and calculated traits. Eight measurements of critical fresh-market grape berry-related traits were evaluated. In this case, the entire cluster was broken down into its different parts, which were peduncle, rachis, pedicel, and berries. Berry-length (BL) was evaluated as the distance between the apical tip and the highest point on the end of each berry. Berry-width (BWI) was measured as the distance between the two extreme points of the equatorial zone of each berry. Berry weight (BWE) was measured and recorded in grams. Firmness (FF) of intact berry (newton, N) was determined using a digital Lutron FR-5120 Fruit Hardness penetrometer equipped with a 2 mm diameter flat probe in the presence of the skin (QA Supplies LLC, Norfolk, VA, USA). The berries were punctured in the lateral, vertical, and diagonal face, and the break-force of berry skin was measured. The scar pattern (SP) was determined on a 50-berry by evaluating the percentage of berries with dry stem scars versus wet scars.

### 4.4. Statistical Analysis

Data of several evaluated traits were collected and analyzed to test the genotype effect using repeated measures of analysis of variance (ANOVA) in SAS (SAS version 9.4, SAS Institute Inc., Raleigh, NC, USA) using PROC GLIMMIX. Means for the analyses were determined using the LSMEANS statement and means separation conducted using the Tukey–Kramer adjusted multiple means comparison test. All data presented as the mean ± SD of 5 biological replicates among three years of experiment (*n* = 15). Principal component analysis (PCA) and hierarchical clustering were carried out using XLSTAT software to examine the grouping of genotypes. PCA was run on the log2-transformed area using the individual variables. Hierarchical clustering was run using the complete linkage method with correlation.

## 5. Conclusions

Overall, the biometrics assessment generated during the current study can be used for evaluating new muscadine advance selections suitable for fresh consumption and wine production, replacing the time-consuming laboratory analyses with intense correlation analysis of traits coordinating diverse quality parameters.

Generally, the diversity in CWE, N.B/C, CC, BL, BWI, BWE, FF, and SP suggested a potent improvement in these traits via breeding. Based on the population analysis, it is important to indicate that the current population did not highlight a particular muscadine genotype that can be promoted into a new cultivar for fresh consumption. The muscadine cultivars Majesty, Onyx, and Supreme are still considered the superior cultivars due to enhanced overall performance of productivity, stability, and particular berry quality attributes.

**Figure 20:**
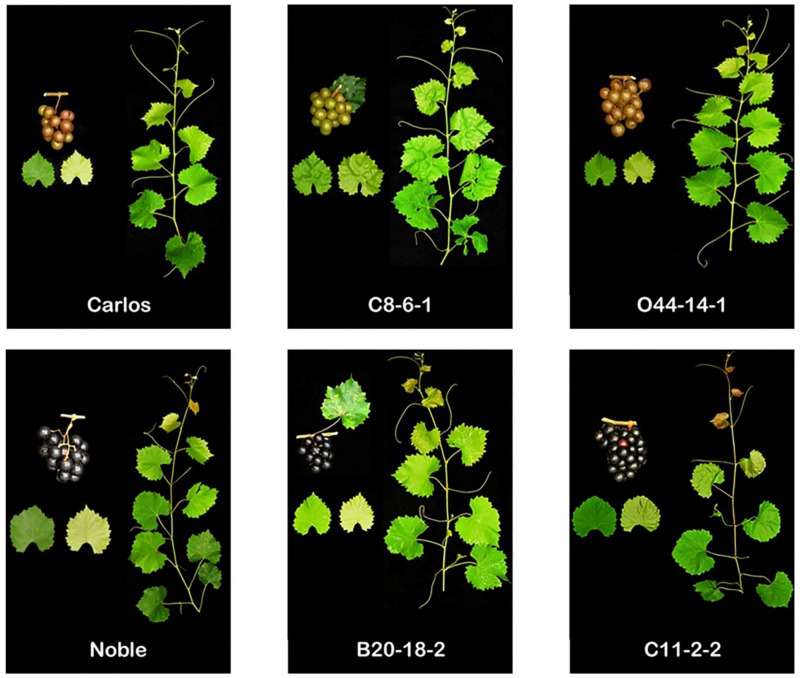
A representative image of leaves, shoots, and cluster of promising muscadine advanced selection genotypes suitable for white (C8-6-1 and O44-14-1) and red (B20-18-2 and C11-2-2) wine production. The standard cultivars Carlos and Noble were used as controls.

By contrast, the current study allowed us to identify several attractive advanced selections suitable for wine production. Their overall performance is considerably better than the current standard cultivars, Carlos and Noble (Table 1 and Table 2). The muscadine genotypes C8-6-1 and O44-14-1 are suitable for white wine production; however, the B20-18-2 and C11-2-2 genotypes are suitable for red wine production (Figure 20). In general, they have superior reproductive characteristics to those of Carlos or Noble. Despite that, they exhibited some minor defects. For instance, the C8-6-1 muscadine genotype produces female flowers, which is not optimal for the growers. Further, both C8-6-1 and C11-2-2 genotypes produce compact clusters. Finally, the B20-18-2 and C8-6-1 berries showed high levels of wet scar pattern.

## Figures and Tables

**Table 1:** Cluster characteristics of muscadine genotypes with high performance to promote into new standard cultivars.

Genotype	Flower	Cluster Wgt. (g)	No. Berries/Cluster	Cluster Compactness	Yield (kg)
Carlos *	Perfect	41.9 ± 1.4	7.6 ± 0.6	6.8 ± 0.8	5.4 ± 0.8
C8-6-1	Female	149.1 ± 3.1	20.8 ± 2.7	28.9 ± 1.5	17.3 ± 1.5
O44-14-1	Perfect	56.4 ± 0.6	13.8 ± 0.4	11.4 ± 1.1	9.9 ± 1.0
Noble *	Perfect	47.8 ± 1.5	12.2 ± 0.4	6.9 ± 0.8	3.9 ± 0.4
B20-18-2	Perfect	61.1 ± 1.1	13.2 ± 1.1	9.2 ± 1.0	11.2 ± 1.2
C11-2-2	Perfect	97.9 ± 5.7	27.6 ± 4.7	18.9 ± 1.3	11.3 ± 1.6
Average **	-	96.9 ± 42.7	11.1 ± 6.8	13.2 ± 6.1	6.6 ± 4.5
Median	-	88.1	8.7	12.2	5.3

* Standard cultivars, Carlos and Noble were used as controls. ** The average is the mean among the population results from 5 biological replicates among three years of the experiment.

**Table 2:** Berry characteristics of muscadine genotypes with high performance to promote into new standard cultivars.

Genotype	Color	Berry Wgt. (g)	Dry Scar (%)	Firmness (N)	No. Seeds/Berry	Wgt. Seeds/Berry (g)
Carlos *	Bronze	6.7 ± 0.4	54 ± 1.1	0.16 ± 0.04	4.0 ± 0	0.34 ± 0.05
C8-6-1	Bronze	8.6 ± 0.5	18 ± 0.9	0.10 ± 0	4.0 ± 0	0.44 ± 0.05
O44-14-1	Bronze	4.6 ± 0.5	72 ± 2.9	0.05 ± 0	3.2 ± 0.4	0.22 ± 0.04
Noble *	Black	4.2 ± 0.3	28 ± 1.1	0.06 ± 0.02	3.8 ± 0.4	0.24 ± 0.05
B20-18-2	Black	5.2 ± 0.3	18 ± 0.7	0.10 ± 0	4.2 ± 0.4	0.27 ± 0.03
C11-2-2	Dark red	3.0 ± 0.3	64 ± 2.6	0.03 ± 0	3.2 ± 0.4	0.28 ± 0.04
Average **	-	12.2 ± 5.6	49.7 ± 22.4	0.12 ± 0.05	3.6 ± 0.7	0.35 ± 0.11
Median	-	11.9	48	0.11	3.6	0.34

* Standard cultivars, Carlos and Noble were used as controls. ** The average is the mean among the population results from 5 biological replicates among three years of the experiment.

## Data Availability

The data presented in this study are available to anyone up to request.

## Supplementary Materials

The following are available online at https://www.mdpi.com/article/10.3390/plants10061067/s1, Table S1: List of used genotypes and flower structure, Figure S1: Representative image demonstrates muscadine-grape cluster structure.
